# Frequency, severity and causes of unexpected allergic reactions to food, a systematic literature review

**DOI:** 10.1186/2045-7022-3-S3-P134

**Published:** 2013-07-25

**Authors:** A Versluis, A Knulst, A Kruizinga, A Michelsen, G Houben, J Baumert, H Van Os-Medendorp

**Affiliations:** 1 Dermatology & Allergology, UMC Utrecht, Utrecht, the Netherlands; 2 TNO, Zeist, the Netherlands; 3 Food Science and Technology Department, University of Nebraska-Lincoln, Lincoln, NE, USA

## Background

Food allergic patients have to deal with their diet. However, confusing labelling terms of precautionary labels can result in risk-taking behaviour. Even those patients that strictly adhere to their diet experience mild but also severe unexpected allergic reactions to food during their life. The aim was of this study was to describe the frequency, severity and causes of unexpected allergic reactions to food in food allergic patients, aged >12 years, in order to improve health care for these patients.

## Methods

A systematic review was carried out. A search was performed by two researchers, in six electronic databases (CINAHL, Cochrane, EMBASE, Medline, Psychinfo and Scopus). The search was performed with keywords relating to the frequency, severity and causes of unexpected allergic reactions to food.

## Results

Eighteen studies met de inclusion criteria; thirteen observational and five qualitative studies. Little is known about the frequency of unexpected reactions. Peanut, tree nuts and milk are the main causal foods. Severe reactions and even fatalities occur, but prevalence data are scarce. Most reactions take place at home, but a significant number also take place when eating at friends or in restaurants. Labelling issues, but also attitude and risky behaviour of patients can attribute to unexpected reactions.

## Conclusion

Prospective studies are needed to get more insight in the prevalence, severity, quantity of unintended allergen ingested and causes of unexpected allergic reactions to food, to be able to optimize strategies to support patients in dealing with their food allergy.

## Disclosure of interest

None declared.

